# Angiotensin-(1-7) protective effects in neurocognitive disorders: molecular mechanisms to therapeutic implications

**DOI:** 10.3389/fphys.2025.1565270

**Published:** 2025-03-27

**Authors:** Lillia C. Lucas, Katherine D. Kimbark, Victoria L. Vernail, Yuval Silberman, Amy C. Arnold

**Affiliations:** Department of Neuroscience and Experimental Therapeutics, Pennsylvania State University College of Medicine, Hershey, PA, United States

**Keywords:** renin-angiotensin system, inflammation, cognition, brain, aging

## Abstract

Cognition broadly refers to the ability to perform mental processes such as learning and memory, attention, emotional awareness, and higher-order thinking. Cognitive deficits can result from the normal aging process or other factors such as disease progression or injury. While the exact etiology is not fully understood, emerging evidence suggests that enhanced inflammatory and oxidative stress processes during aging can dramatically decrease cognitive function in older adults, as well as contribute to the onset and progression of neurocognitive disorders. Current treatments for neurocognitive disorders have limited efficacy and typically focus on symptom attenuation rather than targeting intrinsic pathophysiology. With the rising aging population, there is a critical need to identify novel treatment approaches that target the underlying inflammatory and oxidative mechanisms contributing to neurocognitive disorders. In this regard, the renin-angiotensin system (RAS) may provide an ideal target, as this hormonal system has been implicated in the regulation of inflammatory and oxidative responses to impact cognitive functions. While most research to date has focused on the deleterious role of angiotensin (Ang) II pathways in age-related cognitive decline and neurocognitive disorders, more recent evidence has examined the potential for targeting Ang-(1-7), a protective hormone of the RAS, to counteract these effects. This review highlights emerging evidence showing that activation of Ang-(1-7) pathways reduces inflammation and oxidative stress and may provide a novel target to improve cognitive function and elicit neuroprotection, in the context of both aging and neurocognitive disorders.

## 1 Introduction

Cognition broadly refers to the ability to competently perform various mental processes, including higher-order thinking, perception, memory tasks, attention, and voluntary movement (Bodiga and Bodiga, 2013). Cognitive deficits can result from the normal aging process or other factors such as neurological disease progression and injury. Regardless of the underlying cause, cognitive deficits have been on the rise, particularly in the aging population, and have led to an accumulation in healthcare costs related to treatment and caregiving. Cognitive decline can also occur independent of normal aging processes and is categorized by the Diagnostic and Statistical Manual of Mental Disorders five based on level of severity as delirium, minor neurocognitive disorder, or major neurocognitive disorder (Hugo and Ganguli, 2014; McDonald, 2017). Minor and major neurocognitive disorders are distinguished by the degree of mental decline and the ability to perform daily tasks and live independently. Within these categories, symptoms are further specified into etiological subtypes for disease states such as dementia types, Parkinson’s disease (PD), Huntington’s disease, and traumatic brain injury (TBI), among others (Hugo and Ganguli, 2014; McDonald, 2017).

Most of the current treatment approaches for neurocognitive disorders have demonstrated limited efficacy in improving symptoms and halting disease progression, likely due to their failure to address the underlying pathophysiology of these disorders. Current classes of drugs used to improve symptoms include cholinesterase inhibitors, N-methyl-D-aspartate receptor antagonists, and selective serotonin reuptake inhibitors, which increase cellular communication and protect against excitotoxicity (Hugo and Ganguli, 2014; Arvanitakis et al., 2019; Gouveia et al., 2022). There is evidence these treatments improve cognitive symptoms in certain types of dementia, such as Alzheimer’s disease (AD), but there is mixed evidence of benefit in other dementia types (Arvanitakis et al., 2019; Hugo and Ganguli, 2014). Thus, there is a critical need to identify new approaches that target mechanisms involved in the underlying pathophysiology of cognitive decline in aging and neurocognitive disorders, such as inflammation and oxidative stress.

In this regard, the renin-angiotensin system (RAS) may provide an ideal target, as this hormonal system has been implicated in the regulation of inflammation and oxidative stress as well as in the pathophysiology of cognitive dysfunction and neurocognitive disorders. While most research to date has focused on the deleterious effects of angiotensin (Ang) II pathways in age-related cognitive decline and neurocognitive disorders, more recent evidence has examined the potential for targeting Ang-(1-7), a protective hormone of the RAS, to counteract these effects. This narrative review highlights emerging evidence showing that activation of Ang-(1-7) pathways may provide a novel target to improve cognitive function and elicit neuroprotection, in the context of both aging and neurocognitive disorders. To find relevant articles, we performed a PubMed search from 01 January 1998, through 22 January 2025, reflecting the time frame for Ang-(1-7) discovery through the submission of this review. The search keywords included RAS, Ang, or Ang-(1-7) plus one of the following: aging, cognition, cognitive, neuroprotection, neurodegeneration, neurodegenerative, brain injury, cerebral, memory, or dementia. This primary search was cross-referenced to other databases of scholarly articles, including Web of Science and Google Scholar.

## 2 The aging brain and neurocognitive disorder pathology

**FIGURE 1:**
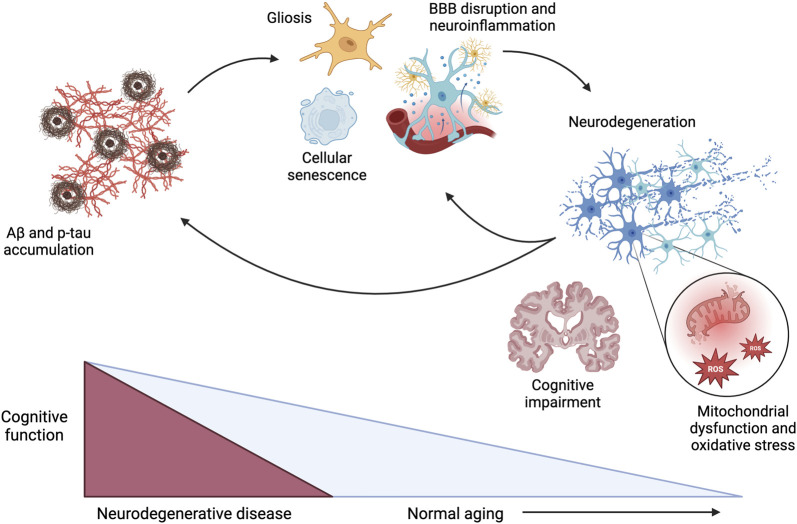
Pathophysiological mechanisms driving cognitive dysfunction during the progression of neurodegenerative disease and normal aging, with acceleration of these mechanisms in the context of neurodegenerative diseases. Aβ: amyloid β; p-tau: hyperphosphorylated tau; BBB: blood-brain barrier; ROS: reactive oxygen species. Created in BioRender. Arnold, A. (2025) https://BioRender.com/x17h875.

The aging process in the brain consists of complex, multifactorial processes. At the core of these processes are the common denominators of inflammation and oxidative stress (Figure 1). Some hypothesized contributors to inflammation and oxidative stress in the brain during aging and disease progression include the accumulation of amyloid-β (Aβ) peptides, hyperphosphorylation of tau protein, mitochondrial dysfunction, damage to cerebral vasculature, cellular senescence, and disrupted insulin signaling. While these individual factors could contribute to inflammation and oxidative stress, they likely play a combined role in the onset and progression of age-related diseases.

Cellular senescence is a pathological hallmark of aging that occurs when cells no longer proliferate but remain metabolically active, causing the release of pro-inflammatory mediators (Finger et al., 2022; Valencia et al., 2022). As such, widespread senescence promotes chronic inflammation, which contributes to cerebrovascular dysfunction and damage; atrophy of astrocytes, oligodendrocytes, and pericytes; disruption of the blood-brain barrier (BBB); and microglial activation (Ahmed and Ishrat, 2020; Finger et al., 2022; Gouveia et al., 2022; Valencia et al., 2022). While Aβ peptides accumulate intrinsically with aging, they are also considered a primary hallmark of neurocognitive impairment beyond normal aging (Costa-Besada et al., 2018; Finger et al., 2022). Aβ causes the release of pro-inflammatory messengers and free radicals and microglial activation, contributing to increased oxidative stress and inflammatory processes (Ahmed and Ishrat, 2020; Finger et al., 2022; Gouveia et al., 2022). Accumulation of Aβ can also contribute to hyperphosphorylation of tau protein, interfering with neurotransmission and causing cellular proliferation and mitochondrial dysfunction (Ahmed and Ishrat, 2020). As a result, the mitochondria produce more reactive oxygen species (ROS), further exacerbating cellular oxidative stress (Dragicevic et al., 2011; Gouveia et al., 2022). As such, this mitochondrial dysfunction is central to the pathogenesis of age-related diseases, dopaminergic neurodegeneration, and neuroinflammation. Finally, disrupted insulin signaling may contribute to neuroinflammation and cognitive impairment. Insulin resistance in the brain is correlated with hyperphosphorylation of tau and elevated glucose levels, further increasing neuroinflammation (Ahmed and Ishrat, 2020; El Idrissi and Alonso, 2022). In support of an association between disrupted insulin signaling and cognitive impairment, diabetes mellitus is associated with cognitive decline, with diabetic patients having a 65% increased risk of developing AD (Arvanitakis et al., 2004). While these collective mechanisms are recognized to contribute to the inflammation and oxidative stress that accompanies cognitive decline in aging and neurocognitive disorders, the optimal approach to directly target these mechanisms to elicit neuroprotection is still under investigation.

## 3 The RAS: Ang II

The RAS is well-recognized for playing a critical role in the regulation of blood pressure and fluid balance and contributing to cardiovascular-related diseases. Beyond these classical cardioregulatory actions, more recent studies have shown that the RAS plays a role in numerous other physiological functions and disease states. Relevant to this review, accumulating evidence shows that overactivation of the Ang II axis of the RAS is associated with the aging process as well as the underlying pathophysiology of neurocognitive disorders. The deleterious effects of Ang II are at least in part mediated by the neuroinflammatory and oxidative stress mechanisms described in Section 2.

### 3.1 Ang II pathways and signaling

**FIGURE 2:**
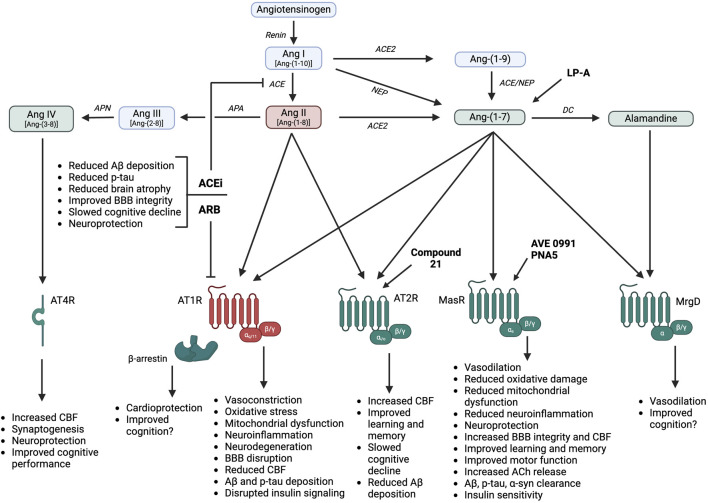
RAS hormones and therapies promote differential cognitive and neuroprotective effects, in part through influences on neuroinflammation, oxidative stress, and vascular function. Ang: angiotensin; AT1R: angiotensin II type 1 receptor; AT2R: angiotensin II type 2 receptor; AT4R: angiotensin IV receptor; MasR: angiotensin-(1-7) Mas receptor; MrgD: Mas-related G protein-coupled receptor; ACE: angiotensin-converting enzyme; ACE2: angiotensin-converting enzyme 2; NEP: neprilysin; DC: decarboxylase; APA: aminopeptidase A; APN: aminopeptidase N; ACEi: angiotensin-converting enzyme inhibitors; ARB: angiotensin receptor blockers; Aβ: amyloid β; p-tau: hyperphosphorylated tau; BBB: blood-brain barrier; CBF: cerebral blood flow; ACh: acetylcholine; α-syn: α-synuclein; LP-A: lactobacillus paracasei releasing angiotensin-(1-7). Created in BioRender. Arnold, A. (2025) https://BioRender.com/a16s721.

Most research to date has focused on the deleterious role of Ang II, also known as Ang-(1-8), in the pathophysiology of age-related cognitive decline and neurocognitive disorders. In this arm of the RAS, the precursor angiotensinogen is cleaved by the enzyme renin to form Ang I, also known as Ang-(1-10) (Figure 2). Ang I is further cleaved by angiotensin-converting enzyme (ACE) to form Ang II. As recently reviewed (Miller and Arnold, 2019), Ang II interacts with receptors localized to peripheral organs (e.g., kidneys, adrenal glands, heart, adipose, skeletal muscle, vasculature) and the central nervous system for various physiological actions. Ang II predominantly binds to type 1 G protein-coupled receptors (AT1R) to induce vasoconstriction, sodium and water retention via aldosterone release, sympathetic activation, mitochondrial dysfunction, and promotion of oxidative stress, inflammation, and immune activation. In addition to G protein-dependent intracellular signaling, activation of AT1R by Ang II can engage intracellular β-arrestin pathways, to promote vasodilation and cardioprotection (Singh et al., 2019). It is important to note that while humans only have one type of AT1R, highly homologous AT1a and AT1b receptor isoforms exist in rodents (Elton et al., 1992). While these isoforms both bind Ang II, they appear to differ in terms of tissue distribution and function (Sasamura et al., 1992; Burson et al., 1994). AT1a receptors are highly expressed in renal, hepatic, adrenal, brain, and cardiac tissues and are thought to primarily mediate Ang II effects on vascular tone and blood pressure. AT1b receptors are highly expressed in the pituitary gland and appear to mediate drinking responses to central Ang II (Davisson et al., 2000). Ang II can also bind to type 2 receptors (AT2R) to produce effects opposite of AT1R activation including cardiovascular, renal, and metabolic protection (Fatima et al., 2021). These receptors, however, are more limited in terms of expression level, affinity, and tissue distribution. Ang II is degraded by aminopeptidase A to form the active metabolite Ang III, also known as Ang-(2-8), and by aminopeptidase N to form Ang IV, also known as Ang-(3-8). Ang III can bind to both AT1R and AT2R, with the relative affinity differing depending on tissue type. Ang IV binds to type 4 receptors (AT4R), which have been postulated to be the transmembrane enzyme insulin-regulated aminopeptidase (Hallberg and Larhed, 2020). In addition to these classical pathways, Ang II can be formed from the renin-independent precursor Ang-(1-12), from Ang I via non-ACE enzymatic pathways such as chymase, and from binding of prorenin to the prorenin receptor (Miller and Arnold, 2019). These alternate formation pathways will not be a focus of this review as there is limited or no data for a role in cognition or neurocognitive disorders.

### 3.2 Ang II molecular pathways in aging and neurocognitive disorders

While circulating Ang peptides are not thought to cross the BBB under normal conditions, Ang peptides, enzymes, and receptors have been demonstrated in circumventricular organs lacking a functional BBB as well as within several brain regions including the brainstem and hypothalamus (Miller and Arnold, 2019). Likewise, local RAS activity has been found in brain regions related to cognitive function including the hippocampus, amygdala, substantia nigra, and striatum (Valenzuela et al., 2016; Jackson et al., 2018; Rukavina Mikusic et al., 2021). Overactivation of Ang II-ACE-AT1R pathways has been implicated in the pathophysiology and progression of both aging and neurocognitive disorders. Ang II promotes cognitive dysfunction via several AT1R-mediated mechanisms including blood pressure elevations, neuroinflammation, oxidative stress, mitochondrial dysfunction, neuron damage, reduced cerebral blood flow, increased Aβ protein precursor mRNA, and BBB disruption (Jiang et al., 2016; Costa-Besada et al., 2018; Wright and Harding, 2019). All of these mechanisms can contribute to disruptions in memory consolidation and retrieval, glutamate neurotoxicity, and increased neuronal Aβ protein levels and tau phosphorylation (Jackson et al., 2018). The observed increase in neuronal Aβ levels and tau phosphorylation is thought to be primarily mediated by the vasoconstrictor effects of Ang II, effectively reducing brain blood flow and preventing clearing of Aβ (Ahmed and Ishrat, 2020; Finger et al., 2022). An increase in brain Ang II activity has also been associated with neuronal degeneration in various brain regions due to increased oxidative stress and mitochondrial dysfunction, exacerbating cognitive dysfunction (Costa-Besada et al., 2018; Abiodun and Ola, 2020).

In addition to an overactivation of Ang II-AT1R pathways, there is a downregulation of Ang II-AT2R and Ang IV-AT4R pathways during aging (Costa-Besada et al., 2018; Wright and Harding, 2019). This results in a “double hit” as these protective pathways improve cognition and elicit neuroprotection to counteract the deleterious effects of AT1R activation. As such, restoration of AT2R signaling via chronic administration of the selective agonist Compound 21 increases spatial memory and learning performance, reduces cognitive decline, increases cerebral blood flow, and reduces neuroinflammation and Aβ accumulation in rodent models of aging and neurocognitive disorders (Jackson et al., 2018; Ahmed and Ishrat, 2020). Ang IV is also critical to memory consolidation and learning and counteracts the inhibitory actions of Ang II on cognitive function via AT4R localized to neurons within the cortex, hippocampus, and basal ganglia. Activation of AT4R by Ang IV elicits neuroprotection, as evidenced by increases in cerebral blood flow, long-term potentiation, and memory consolidation and retrieval in rodent models (Ho and Nation, 2018; Gouveia et al., 2022). For example, intra-carotid Ang IV infusion produced a dose-dependent increase in cerebral blood flow when measured by laser-Doppler flowmetry in anesthetized male Sprague-Dawley rats (Kramár et al., 1997). In terms of long-term potentiation, bath application of an AT4R agonist on hippocampal slices from male Sprague-Dawley rats increased calcium influx, resulting in enhanced synaptic efficiency in hippocampal neurons (Davis et al., 2006). As summarized in a recent systematic review, Ang IV and its analogs, as well as AT4R agonists, improve performance on various cognitive tasks in both normal rodents and rodent models of cognitive deficit (Ho and Nation, 2018). This review identified seven studies showing that acute intracerebroventricular or subcutaneous administration of Ang IV or its analogs improved performance on tests of spatial working memory, object recognition, and passive or conditioned avoidance behavior in normal mice and rats without an induced cognitive impairment. Of note, they identified four additional studies that did not show improvements in inhibitory or passive avoidance or spatial working memory with Ang IV, with two of these studies using intra-hippocampal administration. Only one of these collective studies was conducted in females and showed no effect of subcutaneous Ang IV on object recognition in female mice, with no male comparator. An additional eight studies examined the effects of Ang IV or AT4R agonism in male rodent models of cognitive deficit induced by either central or peripheral scopolamine or mecamylamine administration, with all studies showing improvements in measures of spatial working memory and/or passive avoidance conditioning. This review noted several potential limitations, including that most of these studies did not measure physiological parameters, address the optimal time frame for Ang IV administration or evaluation of outcomes, or blind outcome measurements. Overall, there is substantial evidence supporting that AT2R and AT4R agonism, as well as the administration of Ang IV analogs, can improve cognition in rodent models of aging and neurocognitive disorders.

### 3.3 Clinical implications of targeting Ang II pathways

Given that hypertension is an established risk factor for cognitive decline and dementia, it is not surprising that several classes of antihypertensive drugs have been reported to improve cognition and reduce the risk of dementia and AD. ACE inhibitors (ACEi) and angiotensin receptor blockers (ARBs), which block Ang II formation and activity, respectively, appear to be superior to other classes of antihypertensive drugs in terms of cognition and neuroprotection even independent of blood pressure lowering effects (Ho et al., 2021; Zhou et al., 2024). For example, a meta-analysis by Ho et al. (2021) investigated the effects of ACEi and ARBs on cognitive improvement in elderly hypertensive patients and found these drugs improve cognitive performance by reducing memory decline (Ho et al., 2021). ACEi and ARBs also slow cognitive decline and lower the risk of dementia progression in hypertensive patients with mild cognitive impairment (Deng et al., 2022). Unfortunately, most of these studies did not report the specific medications used by patients within these classes of drugs. That being said, there is at least one study for each of the ARBs currently on the market showing positive effects on cognition, with both BBB-crossing (azilsartan, candesartan, telmisartan, valsartan) and non-BBB-crossing (eprosartan, irbesartan, losartan, olmesartan) drugs improving memory performance and processing speed in patients with AD (Ahmed and Ishrat, 2020; Ouk et al., 2021). This suggests a broad class effect of ARBs on cognition despite potential differences in signaling mechanisms for each specific drug. In terms of ACEi, centrally active drugs (captopril, fosinopril, lisinopril, perindopril, trandolapril, zofenopril) appear more effective in protecting against cognitive decline and dementia risk, compared with non-BBB-crossing drugs (benazepril, enalapril, moexepril, quinapril, ramipril) (Rygiel, 2016; Ho et al., 2021; Ouk et al., 2021). While still controversial, there is also evidence supporting better neurocognitive outcomes with ARBs *versus* ACEi. This is attributed to the ability of ARBs to block the deleterious actions of Ang II at AT1R while allowing for unopposed stimulation of protective AT2R pathways by unbound Ang II (Ahmed and Ishrat, 2020). The mechanisms engaged by these therapies to improve cognition are still under investigation but may include enhancements in cerebral blood flow and neurovascular coupling as well as reductions in oxidative stress, neuroinflammation, endothelial dysfunction, Aβ deposition, tau protein, brain atrophy, and BBB permeability (Ho et al., 2021; Zhou et al., 2024). Of interest, both ACEi and ARBs shift the balance of the RAS to increase circulating levels of Ang-(1-7), a protective hormone that generally opposes Ang II actions, in hypertensive rats and humans when measured by radioimmunoassay methods (Campbell et al., 1991; Kohara et al., 1993; Luque et al., 1996; Benter et al., 2011).

## 4 The RAS: Ang-(1-7)

While initially thought to be an inactive metabolite of the RAS, Ang-(1-7) has emerged over the past few decades as a bioactive hormone that protects cardiovascular, renal, and metabolic functions under normal conditions and in chronic disease states. More recent evidence has shown that Ang-(1-7) pathways are downregulated in conditions associated with cognitive decline, and that restoration of this hormone can engage anti-inflammatory and antioxidant mechanisms to improve cognition in animal models of aging and neurocognitive disorders.

### 4.1 Ang-(1-7) pathways and signaling

As shown in Figure 2, Ang-(1-7) is predominantly formed from the degradation of Ang II by angiotensin-converting enzyme 2 (ACE2), but can also be formed more minorly from the cleavage of Ang I by endopeptidases such as neprilysin (Jackson et al., 2018; Miller and Arnold, 2019; Rukavina Mikusic et al., 2021). Additionally, Ang I can be cleaved by ACE2 to form Ang-(1-9), which is then cleaved by neprilysin or ACE to form Ang-(1-7). The most widely studied physiological actions of Ang-(1-7) include blood pressure lowering, vasodilation, sympathetic inhibition, insulin sensitization, and antioxidant and anti-inflammatory effects (Passos-Silva et al., 2013; Medina and Arnold, 2019). Ang-(1-7) actions have generally been attributed to the activation of Mas receptors (MasR), which are G protein-coupled receptors that are localized to numerous peripheral tissues (e.g., heart, kidneys, liver, adipose, skeletal muscle, blood vessels) and the central nervous system (Vernail et al., 2024). Some recent studies, however, have suggested that Ang-(1-7) may bind to other receptors or that MasR can heterodimerize with other receptors (e.g., AT1R, AT2R, bradykinin B2, endothelin B, dopamine D2) (Bader et al., 2018). The *in vivo* importance and cell and tissue specificity of these interactions remain under investigation. While additional pathways for Ang-(1-7) formation and activity have been more recently discovered including mas-related G-protein coupled receptors (MrgD) and alamandine (Schleifenbaum, 2019), the physiological relevance of these pathways is still under investigation.

### 4.2 Ang-(1-7) molecular pathways in aging and neurocognitive disorders

In addition to cardiovascular-regulatory regions, Ang-(1-7) pathway components have been detected in neurons, astrocytes, mitochondria, and arteries within brain areas related to cognition (Jiang et al., 2016; Hernandez et al., 2021). MasR and ACE2 gene and protein expression have been detected in the hippocampus, amygdala, cortex, hypothalamus, brainstem, basal ganglia, and striatum in mice, rats, non-human primates, and humans using quantitative real-time and digital droplet polymerase chain reaction (PCR), Western blot, and immunolabeling methods (Bodiga and Bodiga, 2013; Costa-Besada et al., 2018; Jackson et al., 2018; Rukavina Mikusic et al., 2021; Gouveia et al., 2022). These protective Ang-(1-7)-ACE2-MasR pathways appear reduced in the brain during aging and neurocognitive disorders as described in detail in subsequent sections.

Emerging evidence shows that restoration of these Ang-(1-7) pathways can attenuate the inflammatory and oxidative stress mechanisms that contribute to cognitive decline in animal models of aging and neurocognitive disorders. Ang-(1-7) has been shown to improve cognition and elicit neuroprotection via several mechanisms, including effects on long-term potentiation, neuroinflammation, oxidative stress, cellular senescence, Aβ and tau levels, and cerebrovascular function in rodents (Hellner et al., 2005; Costa-Besada et al., 2018; Varshney and Garabadu, 2021). A recent study also showed that treatment of cultured astrocytes from the hippocampus of either sex Wistar rat neonates with Ang-(1-7) induces reactive astrogliosis, promotes the secretion of factors into the extracellular environment to enhance the neurotrophic activity of neurons, and confers neuroprotection against glutamate-induced excitotoxicity (Barbosa et al., 2024). The cognitive-enhancing effects of Ang-(1-7) may also involve improvements in mitochondrial dysfunction. MasR immunoreactivity has been colocalized with mitochondrial markers in cultured dopaminergic neurons and glial cells, with MasR and ACE2 protein expression and the Ang-(1-7) peptide detected in pure isolated mitochondria from the rat nigral region (Costa-Besada et al., 2018). Treatment of isolated rat nigral mitochondria with Ang-(1-7) for 24 h inhibited superoxide production induced by activation of mitochondrial AT1R, as well as increased mitochondrial nitric oxide levels, supporting the protective effects of Ang-(1-7) against mitochondrial oxidative stress. Similarly, Ang-(1-7) prevented the deleterious effects of Ang II on mitochondrial dynamics in cultured N27 rat dopaminergic neurons via an anti-inflammatory interleukin-10-mediated mechanism (Quijano et al., 2024). In addition to the brain, Ang-(1-7) has been reported to improve mitochondrial dynamics, ultrastructure, and function in isolated adipocytes and cardiac and renal tissue in male mice (Vargas-Castillo et al., 2020; Chen et al., 2023; Yu et al., 2024).

Overall, Ang-(1-7) pathway components are present in brain regions involved in cognition and are reduced during aging and in neurocognitive disorders. Restoration of Ang-(1-7) levels or signaling improves cognitive function and elicits neuroprotection in rodent models by protecting against the factors known to contribute to central oxidative stress and inflammation that are described in Section 2. As summarized in Table 1 and in subsequent sections, the precise mechanisms engaged by Ang-(1-7) appear to depend on the neurocognitive disease state being studied. Given these findings, Ang-(1-7) is currently being explored as a novel target to improve cognitive function in the context of both aging and neurocognitive disorders.

**TABLE 1:** Summary of cellular and physiological effects of targeting Ang-(1-7) pathways in neurological disorders and diseases.

Disease State	Toxic protein aggregation	Oxidative stress/mito dysfunction	Inflammation	Neuro-degeneration	BBB integrity	CBF	Learning and memory	Motor function
Aging	⇓ Jiang et al., 2016	-	⇓ Buford et al., 2020; Carter et al., 2020	-	⇑ Mi et al., 2021	-	⇑ Mi et al., 2021	-
Dementia	⇓ Jiang et al., 2016; Kehoe et al., 2016	⇓ Varsheny and Garabadu, 2021	⇓ Hay et al., 2019; Jiang et al., 2018	⇓ Duan et al., 2021	⇑ Uekawa et al., 2016	-	⇑ Duan et al., 2021; Smith et al., 2022; Uekawa et al., 2016; Varshney and Garabadu 2021	-
VCID	⇓ Hoyer−Kimura et al., 2021	⇓ Hay et al., 2019	⇓ Hay et al., 2019	-	-	-	⇑ Hay et al., 2017; Hay et al., 2019	-
PD	⇓ Gao et al., 2022	⇓ Rabie et al., 2018	⇓ Bernard et al., 2024	⇓ Duan et al., 2014; Gao et al., 2022	-	-	⇑ Bernard et al., 2024	⇑ Li et al., 2021
MS	-	-	⇓ Lund et al., 2019	⇓ Lund et al., 2019	-	-	-	⇑ Lund et al., 2019
TBI	⇓ Bruhns et al., 2022	-	⇓ Janatpour et al., 2019	⇓ Janatpour et al., 2019; Bruhns et al., 2022	-	-	⇑ Janatpour et al., 2019; Bruhns et al., 2022	-
Non-TBI	-	⇓ Wu et al., 2015	⇓ Regenhardt et al., 2014	⇓ Regenhardt et al., 2013; Regenhardt et al., 2014	⇑ Wu et al., 2015	⇑ Xie et al., 2014	⇑ Xie et al., 2014	-

VCID, vascular contributions to cognitive impairment and dementia; PD: Parkinson’s disease; MS: multiple sclerosis; TBI: traumatic brain injury; ACh: acetylcholine; BBB: blood-brain barrier; CBF: cerebral blood flow; Non-TBI: non-traumatic brain injury (i.e., stroke, cerebral hypoperfusion); mito: mitochondrial. ⇓, decrease; ⇑ increase; -, unknown.

### 4.3 Ang-(1-7) in aging and dementia types

Even during healthy aging, there are often symptoms of neurocognitive decline, such as memory and motor impairments and decreased learning abilities. As previously stated, the Ang-(1-7)-ACE2-MasR axis appears reduced in aging (Jiang et al., 2016; Costa-Besada et al., 2018). In aged male rats, there are reductions in MasR gene expression, ACE2 gene and protein expression and enzymatic activity, and Ang-(1-7) peptide levels in the substantia nigra (Costa-Besada et al., 2018). As evidence of the functional importance of this reduction in protective RAS signaling, Silva et al. (2012) demonstrated that older mice were more prone to oxidative stress and endothelial dysfunction in cerebrovascular arteries, and this effect was exacerbated in ACE2 knockout mice (Silva et al., 2012). Ang-(1-7) pathways are also reduced in rodent models of cognitive decline associated with AD. In the senescence-accelerated mouse prone 8 (SAMP8) mouse model of sporadic AD, Ang-(1-7) peptide levels are reduced in the whole brain of males as measured by ELISA and are inversely correlated with hyperphosphorylated tau in the hippocampus and prefrontal cortex (Jiang et al., 2016; Rukavina Mikusic et al., 2021). Further, clinical studies have shown that circulating Ang-(1-7) levels, as measured by ELISA, are reduced in men and women with mild to moderate AD and correlate with increased white matter abnormalities and diagnosis severity (Ribeiro et al., 2021). Likewise, a study of post-mortem brains from men and women with AD revealed that ACE2 activity was reduced by ∼50% in the mid-frontal cortex, which was inversely correlated with Aβ and phosphorylated tau levels (Kehoe et al., 2016). Thus, decreased Ang-(1-7) pathways have been observed in patients with AD, and appear to correlate with the pathophysiology and severity of disease. While both men and women were included in these clinical studies, sex differences in outcomes were not assessed.

Given this observed reduction in Ang-(1-7) pathways, studies have begun to investigate the potential for targeting Ang-(1-7) in the treatment of age-related cognitive impairment, dementia, and AD. An initial study showed that decreased whole brain Ang-(1-7) peptide levels measured by ELISA correlate with increased pro-inflammatory markers (interleukin-1β, interleukin-6, and TNF-α) in aged male SAMP8 mice (Jiang et al., 2018). In this study, chronic systemic administration of the non-peptide mimetic of Ang-(1-7), AVE0991, attenuated neuroinflammation via MasR-mediated suppression of microglial inflammatory responses. Mi et al. (2021) examined the impact of acute intranasal AVE0991 treatment in aged male rats immediately following laparotomy surgery to mimic the neurocognitive delay displayed by elderly patients post-surgery. They found that surgical trauma decreased MasR protein expression measured by Western blot, and decreased Ang-(1-7) and increased Ang II peptide levels measured by ELISA, in the hippocampus. Treatment with AVE0991 increased MasR protein expression and reduced neuroinflammation in the hippocampus, restored BBB integrity, and improved spatial memory in this model (Mi et al., 2021).

Similarly, in an Aβ-induced male mouse model of AD, treatment with Ang-(1-7) for 14 days improved spatial memory and learning and increased acetylcholine activity in a MasR-dependent manner, suggesting that Ang-(1-7) enhances cognitive performance by improving cellular communication (Varshney and Garabadu, 2021). In male APP/PS1 transgenic mice that model AD progression, intraperitoneal AVE0991 administration for 30 days also rescued spatial cognitive impairments and alleviated neuronal and synaptic damage (Duan et al., 2021). Chronic intracerebroventricular infusion of Ang-(1-7) for 4 weeks also improved cognitive function in the male 5XFAD mouse model of AD by reducing neuroinflammation and BBB dysfunction (Uekawa et al., 2016).

Ang-(1-7) also improves cognitive function in mouse models of vascular contributions to cognitive impairment and dementia (VCID). For example, Hay et al. showed that systemic Ang-(1-7) treatment for 3 weeks improves novel object recognition and spatial memory in male C57BL/6 mice with cognitive impairment due to congestive heart failure, known as the VCID-HF model (Hay et al., 2017). This same group then developed a glycopeptide form of Ang-(1-7), PNA5, which had better brain penetration, a longer half-life, and sustained protective effects compared to Ang-(1-7). They showed that 3 weeks of systemic treatment with PNA5 improves novel object recognition and spatial memory, reduces brain microglia/macrophages, and increases anti-inflammatory cytokines in the male VCID-HF mouse model (Hay et al., 2019). Additional work by Hoyer-Kimura et al. (2021) showed that subcutaneous injection of extended-release PNA5 given post-surgery decreases levels of neurofilament light protein and improves cognitive and memory functions in the male VCID-HF mouse model (Hoyer-Kimura et al., 2021).

Finally, several studies have examined the use of a genetically modified *lactobacillus* paracasei probiotic that promotes the release of Ang-(1-7), referred to as LP-A, to alleviate cognitive impairment in dementia and AD. Carter et al. (2020) tested the effects of LP-A at different dosages in aged male F344BN rats and found that both acute and chronic oral administration increased circulating Ang-(1-7) levels and decreased Ang II levels as measured by ELISA (Carter et al., 2020). Further work by Buford et al. (2020) found that oral LP-A treatment for 4 weeks increases circulating Ang-(1-7) levels measured by ELISA, improves the gut microbiome composition, enhances circulating neurotransmitters related to tryptophan metabolism (serotonin and 2-picolinic acid), and decreases markers of neuroinflammation in the prefrontal cortex (Cox2, interleukin-1β, and TNF-α) of aged male F344BN rats (Buford et al., 2020). Of interest, in this study, while subcutaneous LP-A administration also increased circulating Ang-(1-7) levels, it did not impact neurotransmitter or cytokine parameters, perhaps due to an inability to modulate the gut-brain axis. In a *drosophila* model of AD, 2 weeks of oral LP-A supplementation improved memory performance in males, but not in females (Smith et al., 2022). The authors attributed this differential therapeutic response to potential sex differences in Ang-(1-7) levels and activation of tryptophan metabolism pathways following LP-A administration.

### 4.4 Ang-(1-7) in neurodegenerative diseases

The therapeutic potential of Ang-(1-7) is also being considered in the context of neurodegenerative disorders associated with cognitive decline, such as PD, Huntington’s Disease, and multiple sclerosis. Similar to aging and dementia, protective RAS pathways appear to be downregulated in these neurodegenerative disorders. Li et al. (2021) found reduced Ang-(1-7) levels as measured by ELISA in aged C57BL/6J mice (sex of mice not defined), which was correlated with reduced measures of skeletal muscle function including reduced grip strength and increased number of falls (Li et al., 2021). Additionally, they found that aged ACE2 knockout mice had the lowest overall grip strength, with young ACE2 knockout mice having similar grip strength as older wild-type mice. Similarly, in a male transgenic mouse model of Huntington’s Disease, ACE2 activity measured by ELISA was reduced in the striatum, prefrontal cortex, and hippocampus, while levels of Ang-(1-7) measured by ELISA were reduced in the striatum and hippocampus (Kangussu et al., 2022). In contrast to mouse models, men and women with Huntington’s Disease appear to have higher plasma Ang-(1-7) levels as measured by ELISA compared with healthy controls, perhaps as a protective compensatory response (Kangussu et al., 2022). Together, these findings indicate that reduced expression of Ang-(1-7) pathways is associated with widespread neurological dysfunction.

Restoration of Ang-(1-7) confers neuroprotection and improves motor and cognitive outcomes in animal models of neurodegenerative diseases. In aged ACE2 knockout mice, chronic treatment with Ang-(1-7) improved skeletal muscle motor performance and muscle coordination by promoting cellular glucose metabolism, highlighting a peripheral role of this hormone in motor function (Li et al., 2021). Intra-striatal Ang-(1-7) administration upregulated MasR gene expression and dopamine content in the striatum, increased tyrosine hydroxylase immunoreactivity in the substantia nigra, and decreased proinflammatory and oxidative stress markers in the striatum of male Wistar rats with PD and dopaminergic neuron degeneration produced by 6-OHDA administration (Rabie et al., 2018). In another rat model of PD induced by rotenone exposure, supranigral infusion of Ang-(1-7) for 4 weeks reduced α-synuclein aggregation in the substantia nigra, a hallmark of PD, and increased α-synuclein removal in dopaminergic neurons, relieving PD-induced behaviors in male rats (Gao et al., 2022). In terms of mechanisms involved, recent studies have suggested that the ability of Ang-(1-7) and AVE0991 to attenuate dopaminergic neuronal damage and rescue behavioral impairments in transgenic mouse models of PD involves engagement of microRNA pathways, but this is still under investigation (Duan et al., 2024; Gao et al., 2024). Systemic PNA5 administration for 2 months also attenuated cognitive decline in a chronic progressive male mouse model of PD (Thy1-αSyn mice), at least in part by attenuating neuronal loss and microglial activation in the hippocampus (Bernard et al., 2024). Finally, Ang-(1-7) reduced the severity of multiple sclerosis in male mice by decreasing demyelination, immune infiltration, and axonal loss in the spinal cord in a MasR-dependent manner (Lund et al., 2019).

### 4.5 Ang-(1-7) in brain injuries

Ongoing research is investigating the potential of targeting the Ang-(1-7)-ACE2-MasR axis for treatment of cognitive impairment due to brain injury, both non-traumatic and traumatic. In non-traumatic brain injury, such as cerebrovascular complications, Ang-(1-7) has been shown to confer neuroprotection and improve cognitive outcomes. As previously mentioned, central Ang-(1-7) infusion for 2 weeks protects against ischemia and improves cognitive performance in male Wistar rats with chronic cerebral hypoperfusion (Xie et al., 2014). Central Ang-(1-7) also protected against ischemic stroke and related neurological deficits in male Sprague-Dawley rats via a MasR-mediated inhibition of inflammation and microglial activation in the cerebral cortex (Regenhardt et al., 2013). Ang-(1-7) also improved BBB integrity and reduced hypoxia in rats suffering from cerebral ischemia (Wu et al., 2015). Finally, central Ang-(1-7) infusion for 6 weeks decreased the number of hemorrhages and increased neuronal cell survival in male stroke-prone spontaneously hypertensive rats, leading to prolonged survival rates (Regenhardt et al., 2014).

To date, there are limited preclinical studies that have examined the ability of Ang-(1-7) to attenuate cognitive impairment in the context of TBI. Janatpour et al. (2019) showed that chronic systemic Ang-(1-7) treatment given after controlled cortical impact injury reduced lesion volume, improved spatiotemporal learning and memory deficits, and attenuated microglial and astrocyte activation in the cortex, hippocampus, and thalamus in male TBI mice (Janatpour et al., 2019). In another study, male mice with TBI that were treated with Ang-(1-7) for 5 days post-injury had MasR-dependent improvements in cognitive function, reductions in phosphorylated tau levels, and attenuated neuronal cell loss in the cortex and hippocampus (Bruhns et al., 2022). While providing limited data on cognitive outcomes, these studies show that Ang-(1-7) is involved in the attenuation of factors relevant to the pathophysiology of cognitive dysfunction in the context of TBI.

### 4.6 Limitations of preclinical research on Ang-(1-7) in aging and neurocognitive disorders

While accumulating evidence supports the concept that targeting Ang-(1-7) improves cognition and elicits neuroprotection in animal models of aging and neurocognitive disorders, there are some potential limitations to these studies. First, while most studies have shown positive effects, we identified two studies in which Ang-(1-7) did not improve cognitive outcomes. In one of these studies, chronic systemic Ang-(1-7) infusion for 4 weeks did not improve performance on learning and memory tasks in male spontaneously hypertensive rats, despite lowering blood pressure (Stoyell-Conti et al., 2021). The authors suggested this could be due to an inability of Ang-(1-7) to cross the BBB. In the other study, there was no effect of intra-hippocampal administration of Ang-(1-7) on memory retrieval in male Wistar rats without any induced cognitive deficits (Bonini et al., 2006). While not discussed, this could reflect limitations of direct administration into the hippocampus as well as the use of a cognitively intact model. Second, additional studies may be needed to determine the optimal dose, time course, and mode of administration for Ang-(1-7) and related therapies in aging and neurocognitive disorders. Most of the studies cited used standard pharmacological doses of Ang-(1-7) that are well established in the literature and did not perform dose-response assessments. The time frame for outcome evaluation following Ang-(1-7) administration also varied among studies, with most studies assessing only one time point. In addition, the mode of administration varied and included both systemic (e.g., subcutaneous injections, osmotic mini pump infusion) and central (e.g., intracerebroventricular, administration into discrete brain regions) approaches. While it appears that both peripheral and central administration are effective, the relative efficacy based on the mode of administration remains unclear. Third, there are limitations of the techniques used to quantify Ang peptides, enzymes, and receptors in the cited studies, particularly in brain tissue where endogenous levels may be low. While radioimmunoassay and ELISA remain the most commonly used methods to measure Ang II and Ang-(1-7) peptide levels, the antibodies used for these assays often exhibit cross-reactivity to other Ang peptides (Herrera et al., 2013; Chappell, 2016). In addition, validated antibodies to detect AT1R and MasR protein expression at physiological levels are still lacking (Burghi et al., 2017). While mass spectrometry methods have recently become available to assess RAS components in the circulation and tissues (Rodrigues et al., 2024), this method has not yet been used to examine Ang-(1-7) pathways in specific cognitive brain regions, or to determine the impact of aging or neurocognitive disorders on expression of RAS components. Finally, there are only two studies to our knowledge examining Ang-(1-7) effects on cognition in females, with these studies showing potential sex differences in cognitive outcomes. While one study showed that LP-A improves physical and cognitive function in aged female FBN rats (Hernandez et al., 2023), another showed that LP-A only improved memory deficits in males in a *drosophila* model of AD (Smith et al., 2022). While clinical studies assessing RAS components in neurocognitive disorders have included both men and women, sex differences in these components were not assessed, likely due to the small number of patients in these studies. Given this gap in knowledge, additional research to examine the impact of activating Ang-(1-7) pathways on cognitive outcomes in females is desperately needed.

### 4.7 Clinical implications of targeting Ang-(1-7) pathways

Despite improvements in cognition and neuroprotection with ACEi and ARBs, these therapies often have limited efficacy in terms of blood pressure control and are limited in over 10% of patients due to off-target effects, such as dry cough and, more rarely, angioedema (Arendse et al., 2019; Borghi et al., 2023). Importantly, many of the cardiovascular and metabolic effects of these therapies have been attributed to an increase in endogenous Ang-(1-7) levels (Benter et al., 2011; Loloi et al., 2018). Thus, direct activation of Ang-(1-7) pathways may provide a more targeted approach to improve cognitive outcomes while avoiding the adverse effects seen with traditional RAS-blocking therapies. Indeed, Ang-(1-7) is currently being considered for the treatment of neurocognitive disorders due to its ability to attenuate inflammatory and oxidative responses via multiple mechanisms in animal models. While targeting Ang-(1-7) is of great interest, the translatability of findings has been limited due to the short half-life of this peptide. To overcome this, novel therapies have been developed to more chronically target Ang-(1-7) pathways, which are currently being tested in animal models and early-phase clinical trials for cardiovascular diseases. This includes stable analogs, oral formulations, MasR agonists, and ACE2 activators (Hay et al., 2019; Buford et al., 2020; Carter et al., 2020; Mi et al., 2021; Smith et al., 2022). While some of these novel therapies have been tested in animal models of aging and neurocognitive disorders (e.g., AVE0991, PNA5, LP-A), there are few clinical studies with Ang-(1-7) therapies. Two clinical studies were registered to examine the ability of daily subcutaneous Ang-(1-7) injections to improve cognitive function either in patients undergoing coronary artery bypass surgery or with heart failure, but were suspended due to funding issues and/or slow enrollment (https://www.clinicaltrials.gov, NCT 03252093 and NCT03159988). An additional study is registered to examine the ability of daily subcutaneous Ang-(1-7) injections to improve mental functioning and reduce brain damage in patients with moderate to severe TBI (NCT06282965). Overall, while promising results have been seen in animal models, these findings have not yet been effectively translated to clinical populations. Additional clinical trials are needed to determine the safety and efficacy of targeting Ang-(1-7) to improve cognitive outcomes in patients with cognitive decline due to aging or neurocognitive disorders.

## 5 Conclusion

The current literature provides accumulating evidence to support targeting Ang-(1-7) pathways as a novel approach to improve cognitive outcomes in both aging and neurocognitive disorders. While there are several studies demonstrating that Ang-(1-7) improves cognition and is neuroprotective in animal models by engaging antioxidant and anti-inflammatory mechanisms, clinical studies are needed to confirm these findings in patient populations. The recent development of novel and more stable therapies to target Ang-(1-7) should help to move the field forward in this regard. Additional research is also needed to better understand the mechanisms contributing to the imbalance in the deleterious Ang II *versus* protective Ang-(1-7) axes in the aging process and during the progression of neurocognitive disorders. Potential contributory factors could include comorbid complications such as cardiovascular, gastrointestinal, and metabolic diseases, as well as environmental factors such as nutrition, exposure to toxins, and situational stressors. The precise molecular mechanisms involved in Ang-(1-7) actions should also continue to be explored, particularly related to how these mechanisms might change with sex and in the context of various models of aging and neurocognitive dysfunction. Overall, targeting Ang-(1-7) appears to be a promising approach for the treatment of age-related cognitive decline and neurocognitive disorders.
